# Sustainable Activation of Persulfate Using Corn Cob Biochar for Pesticide Degradation in Wastewater Treatment

**DOI:** 10.3390/molecules30244764

**Published:** 2025-12-13

**Authors:** Tijana Marjanović Srebro, Nina Đukanović, Tajana Simetić, Tamara Apostolović, Jasmina Anojčić, Sanja Mutić, Jelena Beljin

**Affiliations:** Department of Chemistry, Biochemistry and Environmental Protection, Faculty of Sciences, University of Novi Sad, Trg Dositeja Obradovića 3, 21000 Novi Sad, Serbiatamara.apostolovic@dh.uns.ac.rs (T.A.); jasmina.anojcic@dh.uns.ac.rs (J.A.); sanja.mutic@dh.uns.ac.rs (S.M.);

**Keywords:** pesticides, catalytic degradation, biochar, persulfate activation, real surface water

## Abstract

This study investigates the potential of corn cob-derived biochars produced at 400 °C (BC400) and 700 °C (BC700) as heterogeneous catalysts for the degradation of organochlorine pesticides, lindane and β-endosulfan, through persulfate-based advanced oxidation processes (AOPs). BC700 exhibited enhanced degradation performance compared to BC400, likely due to its greater surface area, higher aromaticity, and lower surface polarity. Under optimized conditions (3.0 mM persulfate, pH 7.02, 0.2 g/L biochar), BC700 enabled the removal of up to 94% of β-endosulfan and 82% of lindane within four hours. Quenching experiments suggested different dominant degradation pathways: singlet oxygen (^1^O_2_) appeared to play a key role in lindane degradation, while β-endosulfan degradation likely involved both radical (SO_4_^•−^, HO^•^) and non-radical mechanisms. Reusability tests indicated that BC700 retained catalytic activity for β-endosulfan across multiple cycles, whereas lindane degradation efficiency decreased, possibly due to surface fouling or catalyst deactivation. Experiments conducted in real surface water highlighted the influence of matrix components, with partial inhibition observed for β-endosulfan and an unexpected improvement in lindane removal. These results point to the promise of high-temperature corn cob biochar as a selective and potentially reusable catalyst for AOPs in water treatment, warranting further investigation into regeneration strategies and matrix-specific effects.

## 1. Introduction

Pesticides represent a particularly concerning subgroup of environmental contaminants frequently detected in surface water, groundwater, and even in treated drinking water [1,2]. Conventional drinking water treatment plants (DWTPs) often fail to remove these micropollutants efficiently, allowing their persistence through the treatment process. To contextualize the global scale and persistence of pesticide contamination, recent studies across different regions are summarized in Table 1. As illustrated in Table 1, pesticide contamination has been documented in various water matrices, highlighting its widespread and persistent occurrence across both rural and urban regions. Detection remains a challenge due to the complexity of developing rapid and sensitive analytical techniques, particularly for newly emerging pesticide compounds. Long-term monitoring data reveal that pesticide residues are not only ubiquitous but also temporally persistent, underscoring the need for more effective removal strategies and preventive measures. Collectively, these observations highlight the pressing need for improved technologies and policies focused on the identification, monitoring, and elimination of pesticides from aquatic environments.

Previous studies (Table 1) worldwide have demonstrated the widespread occurrence of pesticides in surface and drinking waters, often at variable concentrations, with certain compounds persisting throughout entire monitoring periods. Seasonal dynamics, rainfall intensity, and agricultural practices further influence pesticide levels, leading to episodic peaks and chronic exposure. High concentrations of herbicides, fungicides, and insecticides have been reported, raising particular concern over pesticide mixtures that may exert synergistic or cumulative toxic effects on aquatic ecosystems. Reported pesticide concentrations generally range from a few ng/L to several hundred ng/L; for instance, carbendazim has been detected up to 4520 ng/L, tebuconazole up to 1071 ng/L, atrazine up to 611 ng/L, and tebufenpyrad at all monitored sites in Greece with a maximum of 330 ng/L. These findings highlight both the frequent occurrence of individual pesticides and the presence of complex mixtures, emphasizing the environmental relevance of pesticide contamination and the need for effective remediation strategies. In response to this challenge, advanced water treatment technologies, particularly adsorption and advanced oxidation processes (AOPs), have gained increasing attention. Among these, persulfate-based AOPs are considered especially promising due to their strong oxidative potential, broad-spectrum reactivity, and minimal formation of harmful by-products [10]. In parallel, biochar (BC) has emerged as a multifunctional material capable of enhancing AOPs efficiency through its dual function as an adsorbent and a catalyst for persulfate (PS) activation. Several studies have demonstrated successful degradation of pharmaceutical contaminants using various BC types, e.g., rice straw [11], coconut shell [12] and red mud-derived BC [13].

Importantly, Simetić et al. (2025) [14] investigated the use of BC derived from hardwood and wheat straw for the degradation of lindane and β-endosulfan in a BC/PS system. Their study confirmed the potential of BC-mediated AOPs for pesticide removal and included experiments in real water matrices, highlighting the importance of testing under realistic environmental conditions. However, the influence of feedstock type remains a critical variable, as BC properties significantly depend on the origin of the biomass and pyrolysis temperature.

Despite growing evidence supporting the application of BC in AOPs, limited data exist on the use of agricultural waste materials such as corn cob as feedstock for BC preparation. Furthermore, the potential of such materials for the removal of persistent organochlorine pesticides has not been fully explored, particularly in terms of mechanistic pathways, reusability, and performance in complex water matrices.

This study aims to address the identified knowledge gap by evaluating the catalytic performance of corn cob-derived BC produced at 400 °C and 700 °C for the degradation of persistent organochlorine pesticides, lindane and β-endosulfan, via PS activation. Corn is widely cultivated in our region, generating substantial quantities of residual biomass, including corn cobs, which are often discarded as agricultural waste. Utilizing this abundant and low-cost material as feedstock for BC production provides an environmentally sustainable solution while promoting the valorization of agricultural residues. Thus, the selection of corn cob as feedstock in this study is motivated not only by its practical availability and regional relevance but also by its documented catalytic potential. Previous studies have shown that BC derived from lignocellulosic residues such as corn cobs develops a well-organized porous structure, enriched oxygen-containing functional groups, and a higher density of defect sites after pyrolysis, all of which are known to enhance PS activation efficiency [15]. In this study, particular emphasis is placed on optimizing key operational parameters (PS concentration, pH, and contact time), identifying the dominant reactive oxygen species (ROS) involved in the degradation process, assessing the reusability of the BC catalyst across multiple cycles, and validating treatment performance in a real surface water matrix. By integrating mechanistic understanding with practical performance evaluation under environmentally relevant conditions, this work aims to explore the potential of low-cost, agricultural waste-derived BC as a sustainable and selective catalyst for pesticide removal in PS-based advanced oxidation processes.

## 2. Results and Discussion

### 2.1. Adsorption by Corn Cob Biochar

**Figure 1:**
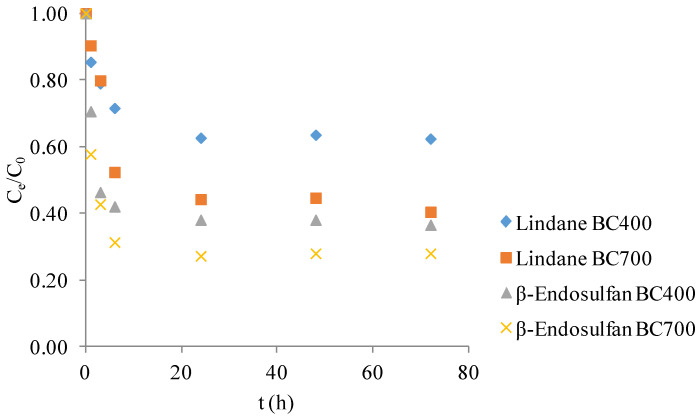
Adsorption kinetics of lindane and β-endosulfan in a binary mixture (each 100 μg/L) on corn cob biochar (0.2 g/L) produced at 400 °C and 700 °C.

Figure 1 presents the normalized concentration (C_e_/C_0_) profiles of lindane and β-endosulfan over 72 h in the presence of corn cob-derived BC produced at 400 °C and 700 °C. These control experiments were conducted in the absence of PS to evaluate the contribution of adsorption as an isolated removal mechanism. A gradual decrease in pesticide concentration was observed during the first 24 h, followed by a plateau, indicating that the system had reached adsorption equilibrium. Overall, pesticide removal efficiency depended on both the pyrolysis temperature of the BC and the type of compound. After 72 h, the estimated removal efficiencies were as follows: β-endosulfan ~63% with BC400 and ~72% with BC700, and lindane ~38% with BC400 and ~59% with BC700. These results confirm that adsorption alone contributed to partial pesticide removal, with BC700 consistently outperforming BC400. The data serve as a baseline for assessing the synergistic effects of catalytic degradation systems involving PS activation.

The adsorption behavior observed here aligns with prior findings by Simetić et al. (2025) [14], where wood- and wheat straw-derived BC also reached adsorption equilibrium within 24 h. In that study, removal efficiencies for lindane ranged from 38–42% with BC produced at 400 °C, and up to 56% at 700 °C. For β-endosulfan, the removal reached 78% with wheat straw BC at 400 °C and 84% at 700 °C. Although corn cob BC exhibited slightly lower removal efficiencies in the present study, the overall trends were comparable, particularly the enhanced performance of BC produced at higher pyrolysis temperatures. These results reinforce the conclusion that both feedstock type and pyrolysis temperature significantly influence adsorption efficiency. Similar conclusions were reported by Kang et al. (2022) [16], who showed that adsorption performance is strongly influenced by the structural evolution of BC during pyrolysis. In our study, differences in adsorption behavior between BC400 and BC700 are consistent with the observed changes in BET surface area and elemental composition, which reflect the degree of carbonization.

### 2.2. Degradation with Persulfate Without Catalyst

**Figure 2:**
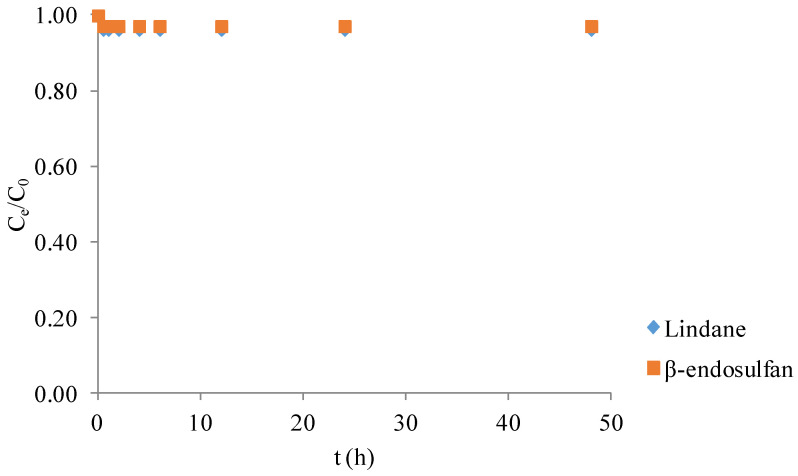
Degradation of lindane and β-endosulfan (each 100 μg/L) by persulfate (3.0 mM) in the absence of biochar at 25 ± 1 °C.

The degradation efficiency of lindane and β-endosulfan by PS alone was less than 4% after 48 h of reaction (Figure 2). This indicates that PS without catalyst addition exhibits minimal oxidative activity toward these pesticides under the tested conditions. The observed low degradation efficiency (<4%) of lindane and β-endosulfan using PS alone is consistent with previous studies [14], which reported poor pesticide removal when PS was applied without catalysts. This highlights the limited capability of PS to generate reactive radicals without activation. Therefore, the presence of a catalyst, such as BC, is essential to activate PS and significantly enhance pesticide degradation through radical formation.

### 2.3. Optimization of Persulfate Concentration in the Biochar-Catalyzed Degradation of Pesticides

**Figure 3:**
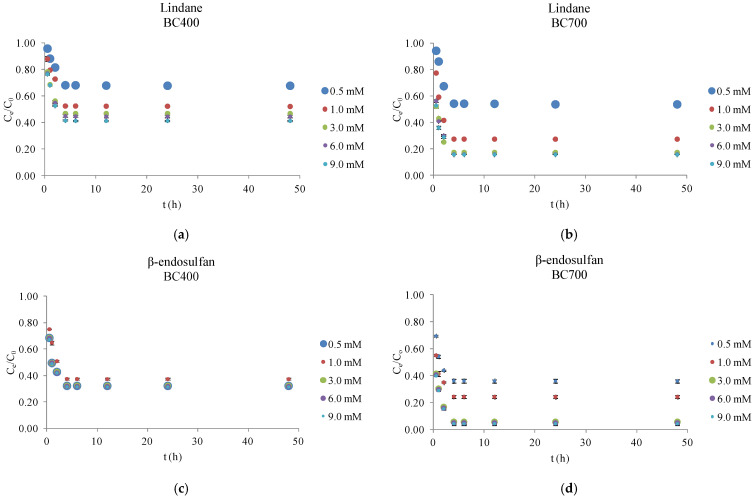
Influence of biochar pyrolysis temperature (400 °C and 700 °C) and persulfate concentration on the degradation of lindane and β-endosulfan. Results are shown as mean ± SD for n = 2. Experimental conditions (**a**–**d**): [biochar = 0.2 g/L; initial pH = 7.02; PS = 0.5–9.0 mM; T = 25 ± 1 °C; stirring = 180 rpm; lindane = 100 µg/L; β-endosulfan = 100 µg/L; volume = 50 mL].

As shown in Figure 3, the degradation efficiency of lindane and β-endosulfan was significantly influenced by both the pyrolysis temperature of the BC and the concentration of PS. BC produced at 700 °C (BC700) exhibited markedly higher catalytic activity across all tested PS concentrations (0.5–9.0 mM) compared to that produced at 400 °C (BC400). This superior performance is attributed to the distinct physicochemical characteristics of BC700, as summarized in Table 2. Elemental analysis revealed that BC700 had a higher carbon content (82.9%) and lower hydrogen (1.61%) and oxygen (1.42%) contents than BC400 (71.9% C, 4.41% H, 5.21% O). These differences are reflected in substantially lower H/C (0.23) and O/C (0.01) molar ratios for BC700, compared to 0.73 and 0.05 for BC400, indicating a higher degree of carbonization and aromaticity. Such structural features are known to enhance π–π interactions with hydrophobic organic contaminants. Additionally, the surface polarity, expressed as the (O + N)/C ratio, was markedly lower for BC700 (0.02) than for BC400 (0.06), suggesting enhanced hydrophobicity. The nitrogen content was also lower in BC700 (0.33%) compared to BC400 (0.64%), which may further contribute to its reduced polarity. Most notably, the BET surface area of BC700 was nearly 15 times higher (306.87 m^2^/g) than that of BC400 (20.87 m^2^/g), providing a significantly greater number of active sites for both PS activation and pesticide adsorption. These findings are consistent with the study by Shen et al. (2021) [17], which demonstrated that a higher specific surface area can enhance catalytic properties. Furthermore, Liu et al. (2020) [18], in their research on tetracycline removal, reported that higher pyrolysis temperatures increase the aromaticity, specific surface area, and pore volume of BC, thereby improving its adsorption characteristics.

Figure 3 illustrates the effect of BC pyrolysis temperature (400 °C and 700 °C) and PS concentration (0.5–9.0 mM) on the degradation of lindane and β-endosulfan, each applied at an initial concentration of 100 µg/L. Corn cob-derived BC was used at a dose of 0.2 g/L. All experiments were conducted in Milli-Q water under controlled laboratory conditions to isolate the influence of the BC/PS system and to identify optimal treatment parameters. Results are reported as mean values of duplicate experiments, with standard deviations below 5%.

Experimental results confirmed that BC700 consistently outperformed BC400 under all tested conditions. At the optimal PS concentration of 3.0 mM, BC700 achieved approximately 82% lindane removal and 94% β-endosulfan removal within 4 h, compared to 53% and 68% for BC400, respectively. Further increases in PS concentration (to 6.0 and 9.0 mM) did not improve degradation, likely due to saturation of reactive sites or radical self-quenching at elevated oxidant levels. Under optimal conditions, the normalized concentration (C_e_/C_0_) of lindane dropped to 0.15 and stabilized, while β-endosulfan decreased to ~0.06, indicating rapid and sustained degradation. In the present study, both pesticides were present simultaneously in a mixed-pollutant system, where synergistic or competitive effects may arise due to interactions with ROS. In such systems, one contaminant may preferentially consume sulfate radicals (SO_4_^•−^), hydroxyl radicals (HO^•^), superoxide radicals (O_2_^•−^), or HSO_5_^−^, potentially accelerating or suppressing the degradation of the other, while some compounds may favor non-radical pathways (electron transfer or singlet oxygen (^1^O_2_)). However, the degradation profiles of lindane and β-endosulfan in this study showed closely aligned removal trends and no significant divergence in C_e_/C_0_ values, indicating that neither strong competition nor enhancement occurred under the tested conditions. This similarity suggests that both pesticides were exposed to a comparable ROS environment and that the presence of one did not markedly inhibit or promote the degradation of the other. These findings emphasize the crucial role of surface area, aromaticity, and hydrophobicity in enabling efficient catalytic performance. These findings align well with previous studies. For instance, Jin et al. (2022) [19] reported that increasing PS concentration above optimal levels did not improve degradation and could lead to radical scavenging, reducing overall reactivity. Similarly, Simetić et al. (2025) [14] observed that PS alone resulted in less than 6% pesticide removal after 48 h, but the presence of high-temperature BC significantly enhanced degradation, especially for β-endosulfan. While this research used BC from hardwood and wheat straw, the trends were consistent, emphasizing the central role of pyrolysis temperature and BC surface properties in catalytic PS activation.

In conclusion, BC700 represents a highly effective and stable catalyst for the degradation of hydrophobic organochlorine pesticides in aqueous environments. The observed plateau at higher PS concentrations underscores the need for careful oxidant optimization to prevent radical quenching and ensure sustainable degradation performance.

### 2.4. Scavenger Experiments for Identification of Reactive Species

**Figure 4:**
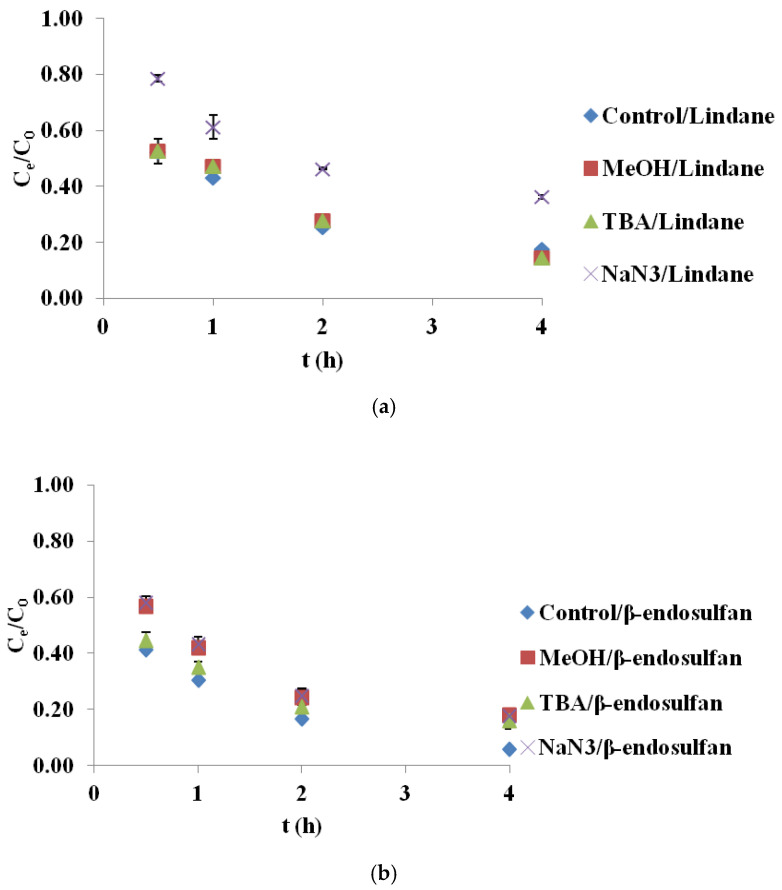
Degradation of (**a**) lindane and (**b**) β-endosulfan in the presence of different scavengers in the BC700/PS system (initial concentration 100 µg/L, PS 3.0 mM, BC700 0.2 g/L, scavengers: MeOH = 100 mM, TBA = 100 mM, NaN_3_ = 100 mM). Results are shown as mean ± SD for n = 2.

To better understand the mechanism of pesticide degradation in the BC/PS system, selective quenching experiments were conducted using methanol (MeOH), tert-butanol (TBA) and sodium azide (NaN_3_) to identify the contribution of ROS. The results are reported as mean values from two measurements, with a standard deviation (SD) of less than 5%. As shown in Figure 4a, the degradation of lindane in the presence of MeOH and TBA was nearly identical to the control, indicating that HO^•^ and SO_4_^•−^ were not the dominant species. However, when NaN_3_ was introduced, a substantial reduction in lindane degradation was observed, confirming that ^1^O_2_ was the primary oxidant involved in this system. In contrast, the removal efficiency of β-endosulfan was significantly affected by all three scavengers (Figure 4b). The addition of MeOH caused a notable decrease in degradation, suggesting the participation of both HO^•^ and SO_4_^•−^. TBA also reduced the degradation efficiency, though to a lesser extent, implicating a role for HO^•^ radicals. The inhibition observed with NaN_3_ points to the contribution of ^1^O_2_ in β-endosulfan oxidation as well. These results imply a combined radical and non-radical mechanism for β-endosulfan degradation, in which all three ROS participate to varying degrees. It should be noted that MeOH, TBA, and NaN_3_ primarily quench solution-phase reactive species, and therefore the possible contribution of surface-bound radicals cannot be fully excluded. Recent studies have shown that PS activation on carbonaceous materials often involves surface-bound SO_4_^•−^ as a dominant pathway, as demonstrated through KI quenching tests and kinetic analysis [20]. In that work, surface-associated reactions were identified as the principal mechanism rather than solution-phase radical oxidation. The use of surface-specific scavengers such as KI or DMSO could therefore provide additional mechanistic insight into whether BC700 promotes surface-mediated PS activation. This is highlighted as an important direction for future investigation.

These findings are consistent with those reported by Simetić et al. (2025) [14], who examined the degradation of lindane and β-endosulfan using BC derived from hardwood (HW700) and wheat straw (WS700) in PS-based systems. In their study, ^1^O_2_ was identified as the dominant species responsible for lindane degradation, as evidenced by the pronounced inhibitory effect of NaN_3_, while MeOH and TBA had minimal impact, mirroring the trends observed in the present BC700/PS system. For β-endosulfan, they reported a more complex degradation pathway involving SO_4_^•−^, HO^•^, and ^1^O_2_, with free radical species playing a more prominent role in the HW700 system and a more balanced contribution in the WS700 system. Despite differences in feedstock origin and pyrolysis temperature, the similarity in observed trends underscores the pivotal role of BC surface chemistry, particularly aromaticity and functional group composition, in governing the dominant degradation mechanisms in PS-activated systems.

### 2.5. Investigation of pH Influence on Persulfate Activation by Biochar

**Figure 5:**
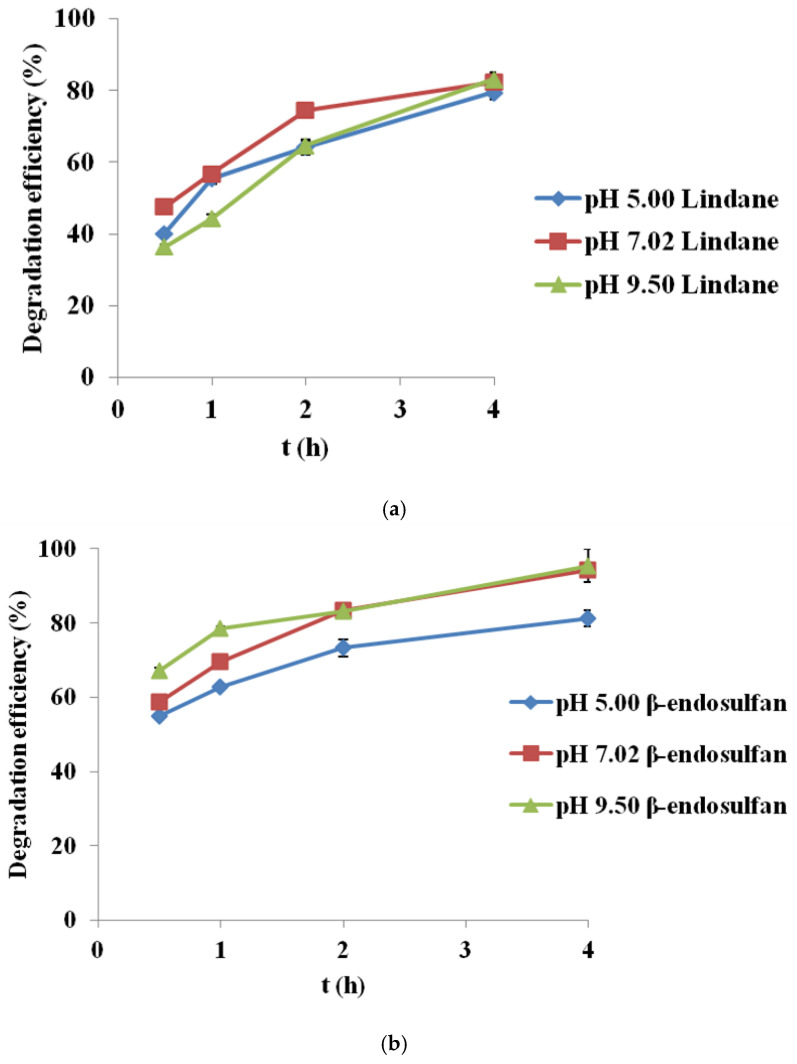
Degradation of (**a**) lindane and (**b**) β-endosulfan at different pH values (initial concentration 100 µg/L, PS 3.0 mM, BC700 0.2 g/L, pH 5.00, 7.02 and 9.50; Results are shown as mean ± SD for n = 2).

Experiments were conducted under optimal conditions (3.0 mM PS, 0.2 g/L BC700, initial pesticide concentration 100 µg/L) at three different pH levels: 5.00, 7.02, and 9.50 (Figure 5). The results are reported as mean values from two measurements, with a standard deviation (SD) of less than 5%.

The results indicate that pH has a moderate impact on the degradation kinetics of both pesticides during the first two hours, while differences diminish after four hours, when removal efficiency becomes high under all tested conditions.

For lindane (Figure 5a), the highest degradation was achieved at neutral pH (82.4% after 4 h), followed closely by alkaline pH (83.1%), while slightly lower efficiency was recorded in acidic conditions (79.5%). In contrast, β-endosulfan (Figure 5b) showed the best performance at alkaline pH (95.4%), followed by neutral (94.3%) and acidic (81.2%) conditions. These results suggest that PS activation by corn cob-derived BC (BC700) remains stable and efficient across a broad pH range. The degradation curves do not start at 0 h because the first measurable sample was collected after complete mixing and stabilization of the BC/PS/pesticide suspension. An initial rapid adsorption phase typically occurs immediately upon BC addition, before the first sampling point, which explains the absence of a 0 h data point.

The observed differences in degradation rates may be related to the pH-dependent speciation of PS. At acidic pH, SO_4_^•−^ formation is likely favored, while at neutral pH both SO_4_^•−^ and HO^•^ radicals could contribute. Under alkaline conditions, PS may decompose more rapidly to HO^•^, although these radicals are more easily scavenged. Additionally, pH can affect the surface charge of BC relative to its pH_pzc_ (~6.3–6.6), potentially influencing pesticide adsorption and contributing to the observed trends.

This pH independence is particularly advantageous for real water treatment applications, where variable pH conditions are common. Our findings are in agreement with Jin et al. (2022) [19], who also demonstrated that BC maintain catalytic activity across different pH environments due to their structural stability and redox-active surface groups. Moreover, Simetić et al. (2025) [14] confirmed the effective degradation of lindane and β-endosulfan in BC/PS systems, regardless of initial pH levels, further supporting the robustness and applicability of such catalytic systems under realistic conditions.

The high and consistent degradation efficiency observed here can be attributed to the reactivity of SO_4_^•−^, which are stable across a range of pH values, in contrast to HO^•^, whose activity is diminished in alkaline media. Additionally, structural properties of BC700, including high surface area, low O/C ratio, and high aromaticity, contribute to maintaining catalytic efficiency regardless of pH. Thus, corn cob-derived BC emerges as a versatile and stable catalyst for PS activation, enabling efficient pesticide removal without the need for pH adjustment.

### 2.6. Possible Mechanism of Persulfate Activation and Pesticide Removal

**Figure 6:**
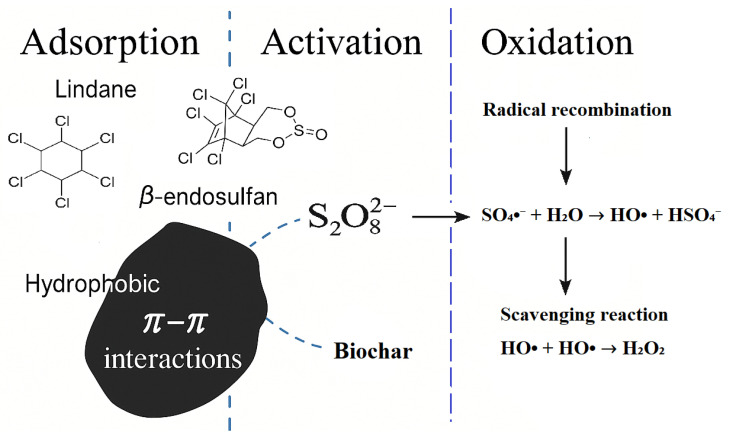
Proposed mechanism of persulfate (PS) activation and degradation of lindane and β-endosulfan in the presence of corn cob-derived biochar (BC). Note: Although the schematic highlights the dominant radical pathways, ^1^O_2_ formation and possible O_2_^•−^/electron-transfer routes are discussed in the text (Section 2.4).

The enhanced degradation of lindane and β-endosulfan in the BC/PS system is attributed to a synergistic mechanism involving both adsorption and catalytic PS activation, as illustrated in Figure 6. The proposed mechanism is consistent with the results of scavenger experiments presented in Section 2.4. BC produced at 700 °C (BC700) offers a large surface area and abundant surface-active sites, enabling efficient pesticide capture and PS decomposition. Due to its aromatic and hydrophobic character, BC700 promotes π–π interactions with the pesticide molecules, facilitating their accumulation on the BC surface. This localized concentration enhances contact between the pesticides and the oxidants. Once adsorbed, PS ions (S_2_O_8_^2−^) are activated by the electron-rich regions of the BC, generating a mixture of ROS, primarily SO_4_^•−^, HO^•^ and ^1^O_2_. These species drive the oxidative degradation of pesticide molecules. At higher PS concentrations, however, scavenging and radical recombination reactions may dominate, leading to decreased efficiency consistent with previously reported findings [14].

Although SO_4_^•−^, HO^•^ and ^1^O_2_ were identified as the main ROS based on scavenger tests, the possible involvement of O_2_^•−^ and electron-transfer pathways cannot be completely excluded, as these mechanisms have been widely reported in BC-activated persulfate systems. Overall, the BC/PS system operates as a BC-mediated AOPs, in which adsorption and catalytic activation are both essential. These results underscore the importance of selecting appropriate feedstock and pyrolysis temperature to tailor BC properties for optimal environmental performance.

### 2.7. Reusability of Biochar

The long-term applicability of BC in PS-based AOPs relies on its catalytic stability and potential for reuse. In this study, the stability of corn cob-derived BC (BC700) was assessed over five consecutive degradation cycles for lindane and β-endosulfan under optimized conditions. After each cycle, the BC was recovered, rinsed with Milli-Q, dried at 60 °C for 12 h and reused in a fresh reaction system.

**Figure 7:**
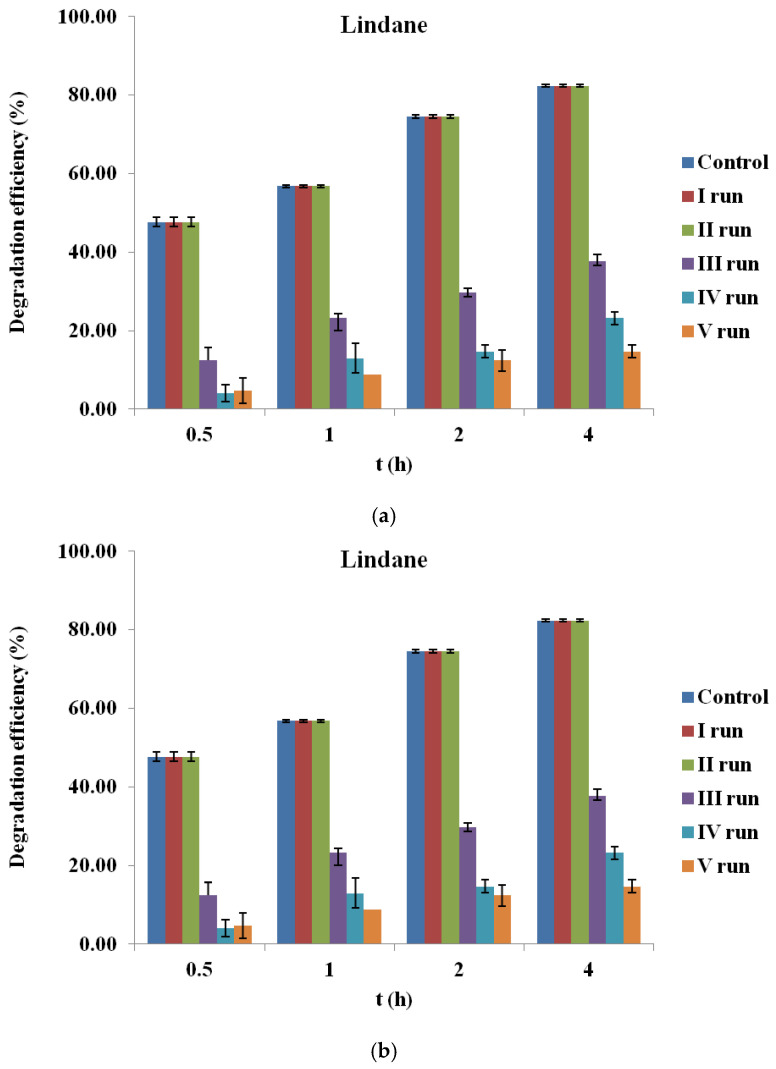
Reusability of corn cob biochar (BC700) in the degradation of (**a**) lindane and (**b**) β-endosulfan over five consecutive cycles (initial concentration 100 µg/L, PS 3.0 mM, BC700 0.2 g/L).

For lindane (Figure 7a), a progressive decline in degradation efficiency was observed over successive cycles. While the first cycle achieved over 70% removal after 4 h, efficiency dropped to below 20% by the fifth cycle. This loss of performance may be attributed to the fouling of active sites by reaction intermediates or degradation products, aggregation of particles, partial deactivation of surface functional groups, or mechanical loss during recovery and filtration. In contrast, β-endosulfan degradation (Figure 7b) remained relatively stable throughout all five cycles, with removal efficiencies consistently exceeding 60%, even in the final cycle. These results suggest that BC700 retains its catalytic functionality more effectively for β-endosulfan than for lindane.

These findings are comparable to those of Simetić et al. (2025) [14], whose evaluated HW700/PS system showed only a 15% decrease in lindane degradation after four cycles, while the WS700/PS system exhibited a pronounced drop (to 43%) by the third cycle. For β-endosulfan, HW700 maintained high stability across all cycles, whereas WS700 experienced a 20–26% efficiency decline after the fourth and fifth cycles.

Compared to those results, the corn cob-derived BC700 examined in this study exhibited slightly lower stability for lindane, but a similarly robust and sustained catalytic performance for β-endosulfan. These findings confirm its potential as a practical and reusable catalyst for pesticide removal in water treatment applications. These interpretations are based on degradation trends and literature reports, as post-reaction characterization of recycled BC700 (e.g., BET, FTIR, XPS) was not performed within the scope of this study.

### 2.8. Experiments in Real Water Matrix

**Figure 8:**
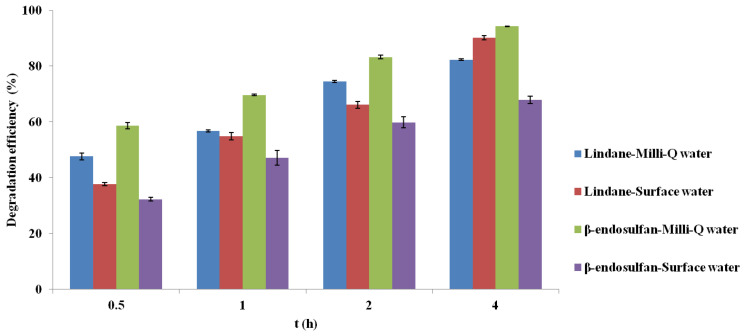
Comparison of lindane and β-endosulfan removal efficiency in deionized and surface water using biochar (700 °C) and persulfate (3 mM) over time. Results are shown as mean ± SD for n = 2).

The results presented in Figure 8 show the degradation of lindane and β-endosulfan (initial concentration 100 µg/L) using corn cob-derived BC pyrolyzed at 700 °C as a heterogeneous catalyst for PS activation (3 mM), with contact times ranging from 0.5 to 4.0 h. Experiments were conducted in real surface water and compared with results obtained in Milli-Q water to assess matrix effects.

Removal of both pesticides was generally more efficient in Milli-Q water, reflecting the influence of real matrix components. Despite similar pH values (7.30 in surface water, 7.02 in Milli-Q water), other factors such as natural organic matter (TOC = 1.98 mg C/L), total nitrogen (0.930 mg N/L), phosphorus (0.361 mg P/L), and ammonia (1.05 mg N/L) likely influenced system efficiency through radical competition or active site blocking. The initial UV254 of the surface water was 0.116 cm^−1^ (Table 3), indicating the presence of a moderate level of UV-absorbing aromatic organic matter typical for natural waters. UV254 was not monitored after treatment, and therefore a quantitative assessment of organic matter removal could not be performed within the scope of this study. This has been acknowledged as a limitation and identified as an important direction for future research. Dai et al. (2023) [21] reported similar findings, showing that humic acids and anionic species can scavenge radicals or deactivate catalytic sites, leading to decreased performance.

Interestingly, lindane exhibited higher removal in surface water after 4 h, possibly due to increased solubility via interactions with organic matter, enhancing its availability for oxidation. In addition, dissolved organic matter containing carbonyl (C=O) functional groups may further promote PS activation through electron-transfer pathways, as reported by Oyekunle et al. (2021) [20], which could also contribute to the enhanced degradation observed in the surface water matrix. In contrast, β-endosulfan showed lower degradation in real water, potentially due to stronger interactions with organic matter that reduce its bioavailability. Wang et al. (2019) [27] also noted dual roles of anions in PS systems, enhancing degradation early but acting as inhibitors at prolonged exposure or higher concentrations due to radical quenching. These findings underscore the complexity of oxidation mechanisms in natural waters and highlight the importance of testing advanced oxidation systems under realistic conditions.

Although the catalytic performance was somewhat diminished in surface water, the system still demonstrated effective pesticide removal, supporting the practical relevance of BC/PS treatments. Future research should aim to elucidate detailed interactions between matrix constituents and reactive species to improve system resilience across diverse water sources.

## 3. Materials and Methods

### 3.1. Chemicals and Reagents

In this study, lindane (PESTANAL^®^ analytical standard, Fluka, Prague, Czech Republic) and β-endosulfan (PESTANAL^®^ analytical standard, Sigma Aldrich, St. Louis, MO, USA) were selected as target analytes. Potassium persulfate (ACS reagent, Sigma Aldrich, St. Louis, MO, USA) was used as the oxidizing agent. Tert-butyl alcohol (ACS reagent, Sigma Aldrich, St. Louis, MO, USA) and sodium azide (Thermo Fisher Scientific, Waltham, MA, USA) were applied as radical scavengers, while sodium thiosulfate pentahydrate (ReagentPlus^®^, Sigma Aldrich, St. Louis, MO, USA) was used to quench residual oxidants. Sodium hydroxide (reagent grade, Sigma Aldrich, St. Louis, MO, USA) and sulfuric acid (Sigma Aldrich, St. Louis, MO, USA) were employed for pH adjustment. Methanol and hexane (HPLC grade, J.T. Baker^®^, Mumbai, India) were used as extraction and analytical solvents. Milli-Q water was obtained using the LABCONCO Water Pro RO/PS Station (Kansas City, MO, USA, Type I quality) and had the following characteristics: dissolved organic carbon < 0.5 mg/L and electrical conductivity of 0.055 µS/cm.

### 3.2. Preparation and Characterization of Biochar

Corn cob biomass used in this study was collected from agricultural land in the Autonomous Province of Vojvodina, Republic of Serbia. The biomass was oven-dried at 60 °C for 24 h to remove residual moisture. Prior to pyrolysis, the dried material was chopped into as small pieces as possible using a knife and a chopper. The chopped biomass was then subjected to pyrolysis in a Nabertherm furnace (MORE THAN HEAT 30–3000 °C, Lilienthal, Germany) under an inert argon atmosphere (flow rate: 80 L/h). The furnace is a closed system, preventing oxygen exposure during pyrolysis. The pyrolysis process was conducted at a heating rate of 10 °C/min up to final temperatures of 400 °C and 700 °C, respectively. The temperatures of 400 °C and 700 °C were selected to obtain BC with distinctly different chemical and structural properties. Lower-temperature pyrolysis (400 °C) typically produces BC with a higher abundance of oxygen-containing functional groups, whereas higher-temperature pyrolysis (700 °C) yields more aromatic, carbonized, and porous structures. Using these two contrasting materials enables systematic evaluation of how the degree of carbonization influences PS activation and catalytic performance. Each sample was held at the target temperature for 1 h and then allowed to cool naturally to room temperature.

After cooling, the solid residue was sieved to remove residual ash, manually ground using an iron pestle, and sieved again to ensure approximately uniform particle size (0.1–1 mm). The resulting BC samples were stored in airtight bags until further use. Two BC materials were produced and labeled BC400 and BC700, corresponding to the applied pyrolysis temperatures. The prepared BC400 and BC700 were characterized by Brunauer–Emmett–Teller (BET) surface area analysis and elemental analysis (CHNS) for carbon, hydrogen, nitrogen, and sulfur content. The surface charge properties were evaluated by determining the point of zero charge (pH_pzc_) following the procedure of Šolić et al. (2020) [28]. The pH_pzc_ values were 6.33 for BC400 and 6.61 for BC700, indicating only minor differences in surface acidity between the two materials (Figures S1 and S2, Supplementary Material). The physicochemical characteristics of the BC are presented in Table 2.

## 4. Control Experiments

### 4.1. Adsorption with Corn Cob Biochar

To isolate the contribution of adsorption in pesticide removal, control experiments were conducted without the addition of potassium persulfate (PS; CAS No. 7727-21-1, Sigma-Aldrich). BC400 and BC700 were added at a dose of 0.2 g/L (10 mg in 50 mL) to Milli-Q water containing 100 µg/L of each pesticide. Experiments were performed under controlled conditions: initial pH 7.02, temperature 25 ± 1 °C, and constant stirring at 180 rpm. The contact time was 1, 3, 6, 24, 48, and 72 h. The samples (50 mL) were withdrawn in given intervals, filtered through a cellulose nitrate filter paper (0.45 µm, Sartorius, Bohemia, NY, USA) and analyzed for residual concentrations.

### 4.2. Degradation with Persulfate Without Catalyst

To evaluate the oxidative capacity of PS alone, additional tests were performed in Milli-Q water containing 100 µg/L of lindane and β-endosulfan, without the addition of BC. PS was applied at 3.0 mM (dose determined as optimal based on experiments described in Section 4.3, “Optimization of Persulfate Concentration in the Biochar Catalyzed Degradation of Pesticides”). Experiments were performed under controlled conditions: initial pH 7.02, temperature 25 ± 1 °C, and constant stirring at 180 rpm. Although the small amount of PS stock solution used in these experiments did not measurably alter the initial pH, it should be noted that PS addition and activation are generally associated with proton generation and gradual acidification, as reported in previous studies [28,29,30]. Therefore, slight pH fluctuations may occur throughout the degradation process. The contact time was 0.5, 1, 2, 4, 6, 12, 24, and 48 h. The samples (50 mL) were withdrawn in given intervals, filtered through a cellulose nitrate filter paper (0.45 µm, Sartorius, Bohemia, NY, USA), and immediately quenched with 0.01 M of NaS_2_O_3_.

### 4.3. Optimization of Persulfate Concentration in the Biochar Catalyzed Degradation of Pesticides

To determine the optimal PS concentration for pesticide degradation, a solution of Milli-Q water containing 100 µg/L lindane (CAS No. 58-89-9, Fluka) and 100 µg/L β-endosulfan (CAS No. 33213-65-9, Sigma-Aldrich) was prepared. For each experiment, 50 mL of this mixed-pollutant solution was used. BC (0.2 g/L of BC400 or BC700) was first placed into clean 60 mL glass vials, after which PS was added at the desired concentration (0.5, 1.0, 3.0, 6.0, or 9.0 mM). The reaction was started by adding the 50 mL pesticide solution to the vial containing BC and PS. The mixture was stirred at 180 rpm and maintained at 25 ± 1 °C. Samples were collected at 0.5, 1, 2, 4, 6, 12, and 24 h, filtered through cellulose nitrate membranes (0.45 µm, Sartorius, Bohemia, NY, USA), and immediately quenched with 0.01 M Na_2_S_2_O_3_. All experiments were performed in duplicate to ensure reproducibility.

### 4.4. Investigation of pH Influence on Persulfate Activation by Biochar

To investigate the effect of initial pH (5.00, 7.02, and 9.50, adjusted using 0.1 M NaOH and 0.1 M H_2_SO_4_) on the degradation of lindane and β-endosulfan, a stock solution of Milli-Q water containing 100 µg/L lindane (CAS No. 58-89-9, Fluka) and 100 µg/L β-endosulfan (CAS No. 33213-65-9, Sigma-Aldrich) was prepared. For each experiment, 50 mL of this mixed-pollutant solution was used. The pH of this pesticide solution was adjusted to the target value (5.00, 7.02, or 9.50) prior to its addition to the reaction vial. BC (0.2 g/L of BC700) and PS (3.0 mM) were then placed in clean 60 mL glass vials, and the experimental run was started by adding the 50 mL pH-adjusted pesticide solution. The catalytic reaction itself is initiated by the interaction between persulfate and the biochar surface. The mixture was stirred at 180 rpm and maintained at 25 ± 1 °C. Samples were collected at 0.5, 1, 2, and 4 h, filtered through cellulose nitrate membrane filters (0.45 µm, Sartorius, Bohemia, NY, USA), and immediately quenched with 0.01 M Na_2_S_2_O_3_. All experiments were performed in duplicate to ensure reproducibility. In this setup, PS acts as the primary initiator of the reaction, while BC serves as a catalyst.

### 4.5. Scavenger Experiments for Identification of Reactive Species

To investigate the dominant ROS involved in the degradation of lindane and β-endosulfan, scavenger experiments were performed using methanol (MeOH, J.T. Baker^®^), tert-butanol (TBA, ACS reagent, Sigma Aldrich), and sodium azide (NaN_3_, Thermo Fisher Scientific, Waltham, MA, USA). MeOH (100 mM) was used as a non-selective quencher for both HO^•^ and SO_4_^•−^, while TBA (100 mM) selectively targeted HO^•^. NaN_3_ (100 mM) was applied to quench ^1^O_2_. Scavengers were added individually to 50 mL reaction mixtures containing 100 µg/L of each pesticide (lindane and β-endosulfan), 3.0 mM PS, and 0.2 g/L of BC700. Experiments were performed under controlled conditions: initial pH 7.02, temperature 25 ± 1 °C, and constant stirring at 180 rpm. The contact time was 0.5, 1, 2, and 4 h. The samples (50 mL) were withdrawn in given intervals, filtered through a cellulose nitrate filter paper (0.45 µm, Sartorius, Bohemia, NY, USA), and immediately quenched with 0.01 M of NaS_2_O_3_. All experiments were performed in duplicate.

### 4.6. Reusability of Biochar

To evaluate the reusability of BC700, five consecutive degradation cycles were performed using the same BC sample. After each cycle, the reaction mixture was filtered (0.45 µm), and the recovered BC700 was thoroughly rinsed with Milli-Q and dried at 60 °C for 12 h before reuse. Each cycle was conducted under identical conditions (initial pesticide concentration 100 µg/L, PS 3.0 mM, BC700 0.2 g/L). Experiments were performed under controlled conditions: initial pH 7.02, temperature 25 ± 1 °C, and constant stirring at 180 rpm. The contact time was 0.5, 1, 2, and 4 h. The samples (50 mL) were withdrawn in given intervals, filtered through a cellulose nitrate filter paper (0.45 µm, Sartorius, Bohemia, NY, USA), and immediately quenched with 0.01 M of NaS_2_O_3_. All experiments were performed in duplicate.

### 4.7. Experiments in Real Water Matrix

To evaluate system performance under realistic conditions, catalytic degradation was carried out in real surface water using the same conditions applied in Milli-Q water (3.0 mM PS, pH 7.02, 0.2 g/L BC700, 180 rpm). The surface water was used without filtration or pretreatment. Key water quality parameters are shown in Table 3. Samples were collected at 0.5, 1, 2 and 4 h, filtered (0.45 µm), and quenched with 0.01 M Na_2_S_2_O_3_. Experiments were performed under controlled conditions: initial pH 7.02, temperature 25 ± 1 °C, and constant stirring at 180 rpm. All experiments were performed in duplicate.

## 5. Analytical Methods

At various intervals after the samples were taken, the concentrations of residual pollutants (lindane and β-endosulfan) were evaluated by gas chromatography coupled with mass spectrometry (GC-MS, Agilent Technologies 7890A Gas Chromatograph/5975C Mass Spectrometer, Santa Clara, CA, USA), employing a DB-5MS capillary column (30 m × 0.25 µm × 0.25 µm, J&W Scientific, Santa Clara, CA, USA) (see the Supplementary Material). The pH of the samples was measured using a pH/ION 735 instrument (Model 04520006; WTW, Weilheim, Germany). The total organic matter content in surface water was evaluated by measuring the total organic carbon (TOC) using a TOC analyzer (Elementar Liqui TOC II, Model 35072028; Elementar, Langenselbold, Germany). UV absorbance at 254 nm was measured with a CINTRA 1010 spectrophotometer (GBC Scientific Equipment, Keysborough, Australia).

## 6. Conclusions

This study presents a comprehensive evaluation of corn cob-derived BC pyrolyzed at 400 °C (BC400) and 700 °C (BC700) for the degradation of lindane and β-endosulfan through PS-based advanced oxidation processes. Although BC400 contributed to partial removal of both pesticides via adsorption, BC700 exhibited significantly higher catalytic efficiency, attributed to its greater surface area, enhanced aromaticity, and lower surface polarity. Optimal degradation occurred under 3.0 mM PS, 0.2 g/L BC dose, and near-neutral pH (7.02).

Reactive species quenching experiments indicated that lindane degradation primarily followed a non-radical pathway mediated by ^1^O_2_, while β-endosulfan degradation involved a mixed mechanism, including SO_4_^•−^, HO^•^, and ^1^O_2_ species.

Reusability tests revealed that BC700 maintained stable removal efficiency for β-endosulfan over five consecutive cycles. However, lindane degradation declined significantly with reuse, likely due to catalyst fouling, loss of active sites, or surface deactivation.

When applied in real surface water, the BC700/PS system remained effective, although matrix constituents such as dissolved organic matter and inorganic ions partially inhibited β-endosulfan removal. Interestingly, lindane degradation improved under these conditions, possibly due to enhanced solubility or altered speciation.

Overall, BC700 emerges as a promising, selective, and partially reusable catalyst for PS activation in water treatment. Its performance under both controlled and environmentally relevant conditions underscores its practical potential. Future research should focus on strategies for catalyst regeneration, improving performance consistency across pollutants, and further clarifying matrix-specific interactions affecting radical reactivity.

## Figures and Tables

**Table 1:** Summary of pesticide occurrence in various water matrices across selected regions.

Region	Time Period	Number of Pesticides Analyzed	Key Findings	Study
Brasil	Not specified	57	Dynamic pesticide occurrence related to agricultural activities. Most frequently detected: clomazone, atrazine, tebuconazole, metconazole, pyrimethanil, carbofuran-3-hydroxide.	[3]
Brasil	2006–2018	17	Carbendazim most abundant (max 4520 ng/L). Tebuconazole detected in 31% of samples (max 1071 ng/L). Atrazine detected up to 611 ng/L.	[4]
Brasil	2011–2014	22	Concentrations ranged from a few to several hundred ng/L. ≥4 compounds detected in >50% of samples. Triazines, triazoles, carbamates, strobilurins, and imidazolinones consistently present. Highlights high water vulnerability and need for monitoring.	[5]
Brasil	2015	12	Monitoring of nine water sources (including drinking water taps) revealed pesticide contamination across different water matrices.	[6]
Spain	2010–2011	50	Organophosphorus and triazine pesticides dominated water and sediments; transformation products exceeded parent compounds. Runoff identified as main source; low flow increased accumulation.	[7]
Spain	Spring 2009–Winter 2010	82	Triazines, propyzamide, and chlorpyrifos most abundant. Seasonal pattern: insecticides peaked in summer, herbicides in winter. Flash floods accounted for >70% of annual input.	[8]
Greece	June 2011–May 2012	34	25 pesticides detected (13 herbicides, 9 insecticides, 3 fungicides). Most frequent: quizalofop-ethyl, trifluralin, pendimethalin. Tebufenpyrad detected at all sites, max 330 ng/L.	[9]

**Table 2:** Physicochemical characteristics of the biochar.

Parameters	BC400	BC700
C (%)	71.9 ± 0.12	82.9 ± 0.42
H (%)	4.41 ± 0.14	1.61 ± 0.08
N (%)	0.64 ± 0.12	0.33 ± 0.01
S (%)	4.09 ± 0.14	4.36 ± 0.82
O (%)	5.21 ± 0.28	1.42 ± 1.34
O/C (molar ratio)	0.05	0.01
H/C (molar ratio)	0.73	0.23
(O + N)/C (molar ratio)	0.06	0.02
BET m^2^/g	20.87	306.87

Values ± standard deviation; Abbreviations: BC produced at 400 °C—BC400 and 700 °C—BC700.

**Table 3:** Characteristics of the surface water.

Parameter	Unit	Result	Method
pH	-	7.30	[22]
TOC ^a^	mg C/L	1.98	-
COD ^b^	mg O_2_/L	18.5	[23]
UV_254_	cm^−1^	0.116	[23]
Total Nitrogen	mg N/L	0.930	[24]
Total Phosphorus	mg P/L	0.361	[25]
Ammonia	mg N/L	1.05	[26]

Abbreviation: ^a^ TOC-Total Organic Carbon; ^b^ COD-Chemical oxygen demand.

## Data Availability

The original contributions presented in this study are included in the article/Supplementary Material. Further inquiries can be directed to the corresponding author(s).

## Supplementary Materials

The following supporting information can be downloaded at: https://www.mdpi.com/article/10.3390/molecules30244764/s1, Figure S1: Determination of the zero point charge (pH_pzc_) for corn cob biomass pyrolyzed at 400 °C; Figure S2: Determination of the zero point charge (pH_pzc_) for corn cob biomass pyrolyzed at 700 °C.
