# Supplementary material for: Sustainable Activation of Persulfate Using Corn Cob Biochar for Pesticide Degradation in Wastewater Treatment
Source: Molecules. 2025 Dec 13;30(24):4764. doi: 10.3390/molecules30244764 (PMC12735698; doi:10.3390/molecules30244764)
Supplement: Supplementary file 1 [file molecules-30-04764-s001.zip › molecules-4028073-supplementary.pdf]

Sustainable Activation of Persulfate Using Corn Cob Biochar for Pesticide Degradation in Wastewater Treatment

Tijana Marjanović Srebro, Nina Đukanović*, Tajana Simetić, Tamara Apostolović, Jasmina Anojčić, Sanja Mutić and Jelena Beljin

University of Novi Sad, Faculty of Sciences, Department of Chemistry, Biochemistry and Environmental Protection, Trg Dositeja Obradovića 3, 21000 Novi Sad, Republic of Serbia
* Corresponding author, e-mail: nina.djukanovic@dh.uns.ac.rs

The zero point of charge

The point of zero charge (pH_{pzc}) of biochar (BC) represents the pH value at which the sum of the positive and negative charges on the material's surface are equal (Figure S1 and Figure S2). The pH_{pzc} of the material was determined using the salt addition method, based on the change in the initial pH value of the inert electrolyte solution influenced by the analyzed material. The determination of the pH_{pzc} for BC was optimized for this study following the procedure described in the literature by Šolić et al. (2020). The pH_{pzc} of the material was determined from the curve of the final pH value (pHf) versus the initial pH value (pHi), where pH_{pzc} corresponds to the value at which pHi equals pHf.

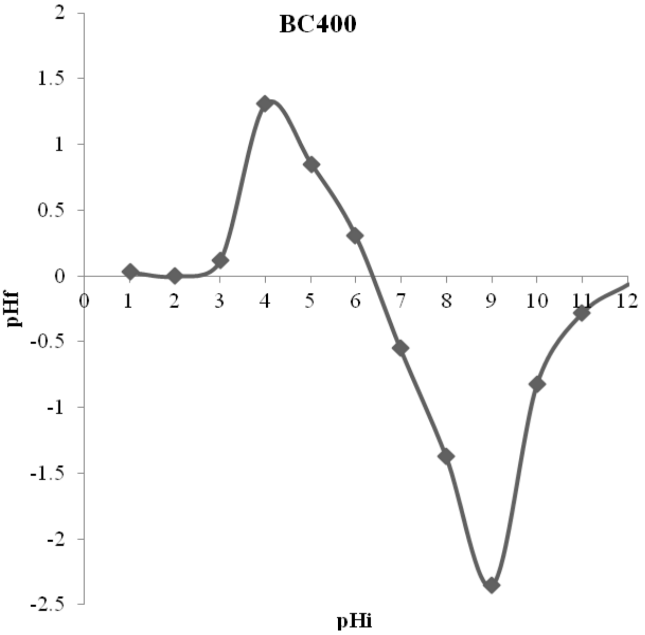

Figure S1. Determination of the zero point charge (pH_{pzc}) for corn cob biomass pyrolyzed at 400 °C.

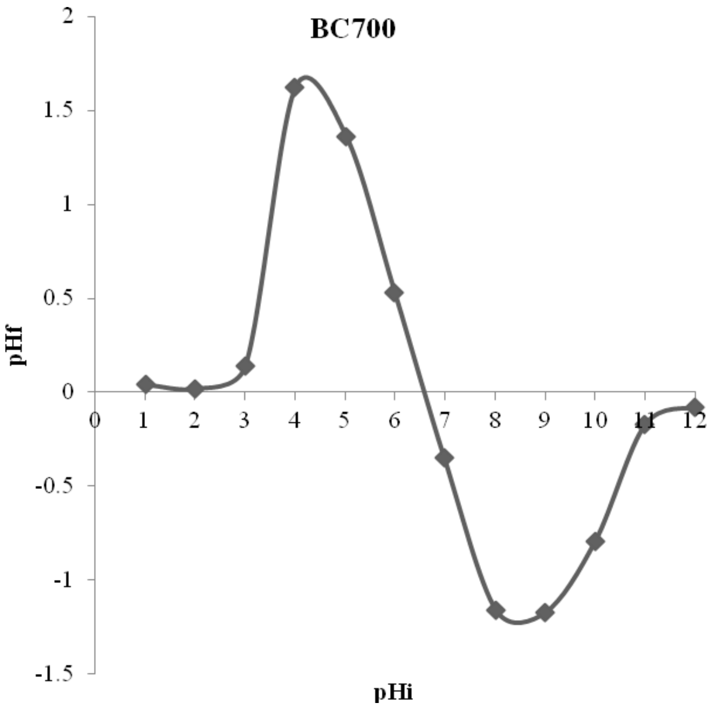

Figure S2. Determination of the zero point charge (pH_{pzc}) for corn cob biomass pyrolyzed at 700 °C.

Analytical method

GC-MS

The chromatographic conditions were as follows: The oven was initially set to 50°C for 1 minute, then ramped to 300°C at a rate of 15°C/min (held for 2 minutes). The ion source temperature was maintained at 230°C, while the quadrupole temperature remained at 150°C throughout the analysis in electron impact mode (70 eV). The transfer line temperature was set to 280°C, with a solvent delay of 3 minutes. A splitless injection was used with a 2 μL injection volume. The analysis was conducted in SIM/SCAN mode. For lindane, the quantification ion was m/z 181, with confirmation ions at m/z 123 and 219. For β-endosulfan, the quantification ion was m/z 241, with confirmation ions at m/z 195 and 339.

References

Šolić, M.; Maletić, S.; Kragulj Isakovski, M.; Nikić, J.; Watson, M.; Kónya, Z.; Tricković, J. Comparing the Adsorption Performance of Multiwalled Carbon Nanotubes Oxidized by Varying Degrees for Removal of Low Levels of Copper, Nickel and Chromium (VI) from Aqueous Solutions. *Water* **2020** 12 ,723. https://doi.org/10.3390/w12030723
